# IRF4 and IRGs Delineate Clinically Relevant Gene Expression Signatures in Systemic Lupus Erythematosus and Rheumatoid Arthritis

**DOI:** 10.3389/fimmu.2018.03085

**Published:** 2019-01-07

**Authors:** Javier Rodríguez-Carrio, Patricia López, Mercedes Alperi-López, Luis Caminal-Montero, Francisco J. Ballina-García, Ana Suárez

**Affiliations:** ^1^Area of Immunology, Department of Functional Biology, Faculty of Medicine, University of Oviedo, Oviedo, Spain; ^2^Instituto de Investigación Sanitaria del Principado de Asturias (ISPA), Oviedo, Spain; ^3^Bone and Mineral Research Unit, REDinREN del ISCIII, Hospital Universitario Central de Asturias, Oviedo, Spain; ^4^Department of Rheumatology, Hospital Universitario Central de Asturias, Oviedo, Spain; ^5^Department of Internal Medicine, Hospital Universitario Central de Asturias, Oviedo, Spain

**Keywords:** interferon, IFN signature, autoimmunity, systemic lupus erythematosus, arthritis, biomarker

## Abstract

**Introduction:** Overactivation of the type I interferon (IFN) signature has been observed in several systemic autoimmune conditions, such as Systemic Lupus Erythematosus (SLE) or Rheumatoid Arthritis (RA). Impaired control of Interferon-Responding Genes (IRGs) expression by their regulatory mechanisms, including Interferon Regulatory Factors (IRFs), may underlie these findings and it may explain the heterogeneity observed among these conditions. In the present study we aimed to evaluate the associations between IRF4 gene expression and those of IRGs in SLE and RA patients to gain insight about its links with the IFN signature as well as to explore the potential clinical relevance of these associations.

**Methods:** The gene expression of IRF4 and IRGs (IFI44, IFI44L, IFI6, and MX1) in peripheral blood was analyzed in 75 SLE patients, 98 RA patients, and 28 healthy controls. A group of 13 biological-naïve RA patients was prospectively followed upon TNFα-blockade. The associations among IRF4 and IRGs were evaluated by principal component analyses (PCA), correlations and network analyses. Publicly available datasets were used for replication.

**Results:** A broad activation of IRGs was observed in autoimmune patients, although certain heterogeneity can be distinguished, whereas IRF4 was only upregulated in RA. The differential expression of IRF4 in RA was then confirmed in publicly available gene expression datasets. PCA revealed different associations among IRF4 and IRGs in each condition, which was later confirmed by correlation and network analyses. Cluster analysis identified 3 gene expression signatures on the basis of IRF4 and IRGs expression which were differentially used by SLE and RA patients. Cluster III was associated with markers of disease severity in SLE patients. Cluster II, hallmarked by IRF4 upregulation, was linked to clinical stage and mild disease course in RA. TNFα-blockade led to changes in the association between IRF4 and IRGs, whereas increasing IRF4 expression was associated with a good clinical outcome in RA.

**Conclusions:** The differential expression of IRF4 and IRGs observed in SLE and RA can delineate gene expression signatures associated with clinical features and treatment outcomes. These results support a clinically-relevant phenomenon of shaping of the IFN signature by IRF4 in autoimmune patients.

## Introduction

The type I interferons (IFNs) are pleiotropic mediators that play a critical role as regulators of innate and adaptive immune responses (1, 2). Signaling through the type I IFN pathway leads to an increased expression of several IFN-responding genes (IRGs). This global expression profile has been termed as the “IFN signature” (3). There is a compelling body of evidence linking the type I IFNs and the presence of the IFN signature to systemic autoimmune conditions in peripheral blood and target tissues (4–6). Either as biomarkers or as disease targets, several studies have been focused on the role of IRGs and the IFN signature in these rheumatic conditions (7). The identification of biomarkers to assist in patient stratification and therapy response is of upmost relevance in these complex conditions, in order to resolve the clinical heterogeneity that hallmarks these diseases (8, 9). This is especially important for decision-making regarding biological treatments, due to their high costs and moderate clinical response in unselected patient populations (10–13).

Type I IFN production is tightly controlled at the gene expression level in a highly ordered process regulated by multiple transcription factors (14). Then, the aberrant IRG expression in autoimmunity may be caused, at least in part, by an impaired activity of their regulatory factors. However, the mechanisms underlying the abnormal triggering and perpetuation of the type I IFN signature in these conditions are poorly characterized. In recent years, the role of Interferon Regulatory Factors (IRFs) has emerged. IRFs are a family of transcription factors that modulate immune responses through various molecular events related to the IFN signaling pathway (15, 16). Among IRFs, IRF1, IRF3, IRF5, and IRF7 have been previously demonstrated to act as regulators of type I IFNs and IRGs transcription (14). IRF1 was the first family member discovered to activate type I IFN gene promoters (17), although further studies found that type I IFN signaling can be observed in Irf1^−/−^ mouse models (18). Later, IRF5 was linked to the expression of type I IFNs. Indeed, gene variants at the IRF5 loci, which are related to autoimmune disease susceptibility, were found to correlate with type I IFN signature (19). Nevertheless, further studies suggest that IRF5 is dispensable for IRGs induction (20). In recent years, IRF3 and IRF7 have been also related to IRGs responses (21–23), acting as negative regulators, this effect being related to the NFkB pathway (24). However, a recent study has challenged this observation (24). Importantly, by targeting IRF3 and IRF7 signaling, only a partial effect on type I IFNs was observed, hence suggesting that additional mediators could be involved (24). Overall, although there is some evidence that IRFs can modulate the IFN signature, the current evidence is scarce. Nevertheless, despite less attention has been paid in early studies, the potential involvement of other family member, the IRF4, has emerged. More importantly, whether these molecular events play any role in the context of autoimmunity remains unknown.

IRF4 is required for proper maturation and differentiation of immune cells (25, 26). IRF4 is expressed in dendritic cells, monocytes/macrophages, granulocytes and B-cells (27), all cell subsets relevant for IFN signature in autoimmunity (28). Moreover, IRF4 loci has been found to be associated with genetic susceptibility to systemic autoimmune diseases (29, 30). Of note, IRF4 has been also related to NFkB pathway (31). Additionally, IRF4 has been revealed to interact with MyD88, an adaptor protein crucial for the activation of IRGs (32).

All these lines of evidence point to IRF4 as a relevant player for IRGs activation and thus, IFN signature in autoimmunity. Taken all these ideas into account, we hypothesize that IRF4 activation could be related to IRGs expression in systemic autoimmune conditions and that different gene expression signatures may be identified on the basis of their associations. Thus, in the present study we aimed to assess the IRF4 gene expression in SLE, RA patients and HC in order to evaluate (i) its association with IRGs expression in these conditions, (ii) the clinical relevance of IRF4 and IRGs in each condition, and (iii) the changes in IRF4 expression upon TNFα-blockade.

## Materials and Methods

### Ethical Approval

The study was approved by the Institutional Review Board (Comité de Ética de Investigación Clínica del Principado de Asturias, reference PI16/00113) in compliance with the Declaration of Helsinki. Written informed consent was signed from all study subjects prior to study entry.

### Patients and Controls

Our study involved 75 SLE patients [age median 48.40 (range 27–75) years, 70 women], 98 RA patients [age median 52.93 (range 22–87) years, 79 women] and 28 age- and gender-matched healthy volunteers (HC) [age median 49.38 (range 35–60) years, 20 women] recruited from the same population. Additionally, a group of 13 biologicals-naïve RA patients [12 women, age median age 43 (range 30–65), DAS28 5.08(1.93), 38.5% RF+, 46.1% ACPA+], candidates for TNFα-blockers was recruited and prospectively followed up for 3 months. A blood sample was collected from all study subjects by venipuncture. In the prospective study, a blood sample was obtained before (baseline, BL) and 3-months after the initiation of the TNFα-blockade therapy (post-treatment, PT). SLE patients were recruited from the outpatient clinic of the Autoimmune Disease Unit [Department of Internal Medicine, Hospital Universitario Central de Asturias (HUCA)] and fulfilled the American College of Rheumatology (ACR) revised criteria for the SLE classification (33). RA patients were enrolled from the Department of Rheumatology (HUCA) and fulfilled the 2010 ACR/EULAR classification criteria (34). A complete clinical examination, including disease activity score calculation [SLE Disease Activity Index (SLEDAI) or Disease Activity Score 28-joints (DAS28), respectively] was performed on all patients during the clinical appointment at their respective departments. Information on further clinical features, including disease-related autoantibodies and treatments (received during the previous 3 months before sampling) were registered from medical records. RA patients recruited at onset and not being previously exposed to treatments were classified as very early RA (VERA). The clinical response of RA patients upon TNFα-blockade was evaluated by EULAR criteria (35). Patients exhibiting a good response (R) were compared to those with moderate or no response (NR).

### RNA Isolation and RT-PCR

Blood samples were immediately processed after collection as previously described (36). Whole blood was mixed with RNA Stabilization Reagent for Blood/Bone Marrow (Roche, Germany) for stabilization and stored at −20°C, in compliance with the instructions provided by the manufacturer. Next, samples were thawed at room temperature and mRNA was isolated using the mRNA Isolation Kit for Blood/Bone Marrow (Roche), according to the protocol provided by the manufacturer. Reverse Transcription (RT) was performed using a High-Capacity cDNA Reverse Transcription Kit (Applied Biosystems).

### Gene Expression Assays

A number of IRG was selected from previous studies in the field using peripheral blood (37–41) and later validated in a factor analysis as those that best reflect the global IFN signature (42, 43). Gene expression was evaluated by Real-Time PCR with pre-designed TaqMan Gene Expression Assays (Applied Biosystems, Germany) for the following genes: IRF4 (interferon regulatory factor 4, reference Hs00180031_m1), IFI44 (interferon induced protein 44, reference Hs00197427_m1), IFI44L (interferon induced protein 44 like, reference Hs00915292_m1), MX1 (MX dynamin like GTPase 1, reference Hs00895608_m1), and IFI6 (interferon alpha inducible protein 6, reference Hs00242571_m1). Reactions were performed in TaqMan Gene Expression Master Mix (Applied Biosystems). Real-Time quantitative PCR was performed in an ABI Prism HT7900 (Applied Biosystems) instrument and Ct values were analyzed with the software SDS 2.3. All samples were assayed by triplicate and the average was used. Expression level was evaluated by the 2^−Δ*Ct*^ method, using the GAPDH gene expression as housekeeping to normalize Ct values. The expression levels were log-transformed and Z-scores were calculated for each gene from the distribution observed in the whole population.

### Statistical Analysis

Continuous variables were summarized as median (interquartile range), whereas *n*(%) was used for categorical ones. Differences among groups were analyzed by Kruskal-Wallis (with Dunn-Bonferroni correction for multiple comparisons if significant differences were observed), Wilcoxon test for paired analises or chi-squared tests, according to the distribution of the variables. Correlations were assessed by Spearman ranks' test. Principal Component Analysis (matrix correlation method) was performed with the individual gene expression data and biplots were generated to evaluate the associations among individual genes. Correlograms and network analyses were built to analyze the correlations among genes as well as to visualize the associations among them in the different conditions. A cluster analysis was performed based on squared euclidean distances and Ward's Minimum Variance Method to identify clusters minimizing the loss of information. The R package *heatmap.2* was used to generate the corresponding heatmap. A Correspondence Analysis was used to explore the simultaneous associations among categorical variables (clusters identified vs. disease groups). Since important differences in sizes were observed, the weighted chi-square distance was selected. For the validation of our results, gene expression datasets were downloaded from the publicly available NCBI Gene Expression Omnibus (GEO) repository (44). First, IRF4 expression was checked to be differentially regulated in the patient groups using the GEO2R tool (using *GEOquery* and *limma* R packages) and the corresponding adjusted *p*-value [multiple testing and false discovery rate corrections by the Benjamini & Hochberg method (45)] was calculated. Next, target data were downloaded and presented in graphs (analysis by conventional tests). A *p* < 0.050 was considered as statistically significant. Statistical analyses were performed in SPSS 22.0 (IBM SPSS, NY, USA), R 3.3.1 (R Project) and GraphPad Prism 5.0 (La Jolla, CA, USA) for Windows.

## Results

### IRF4 and IRGs Expression in SLE and RA Patients

**Figure 1:**
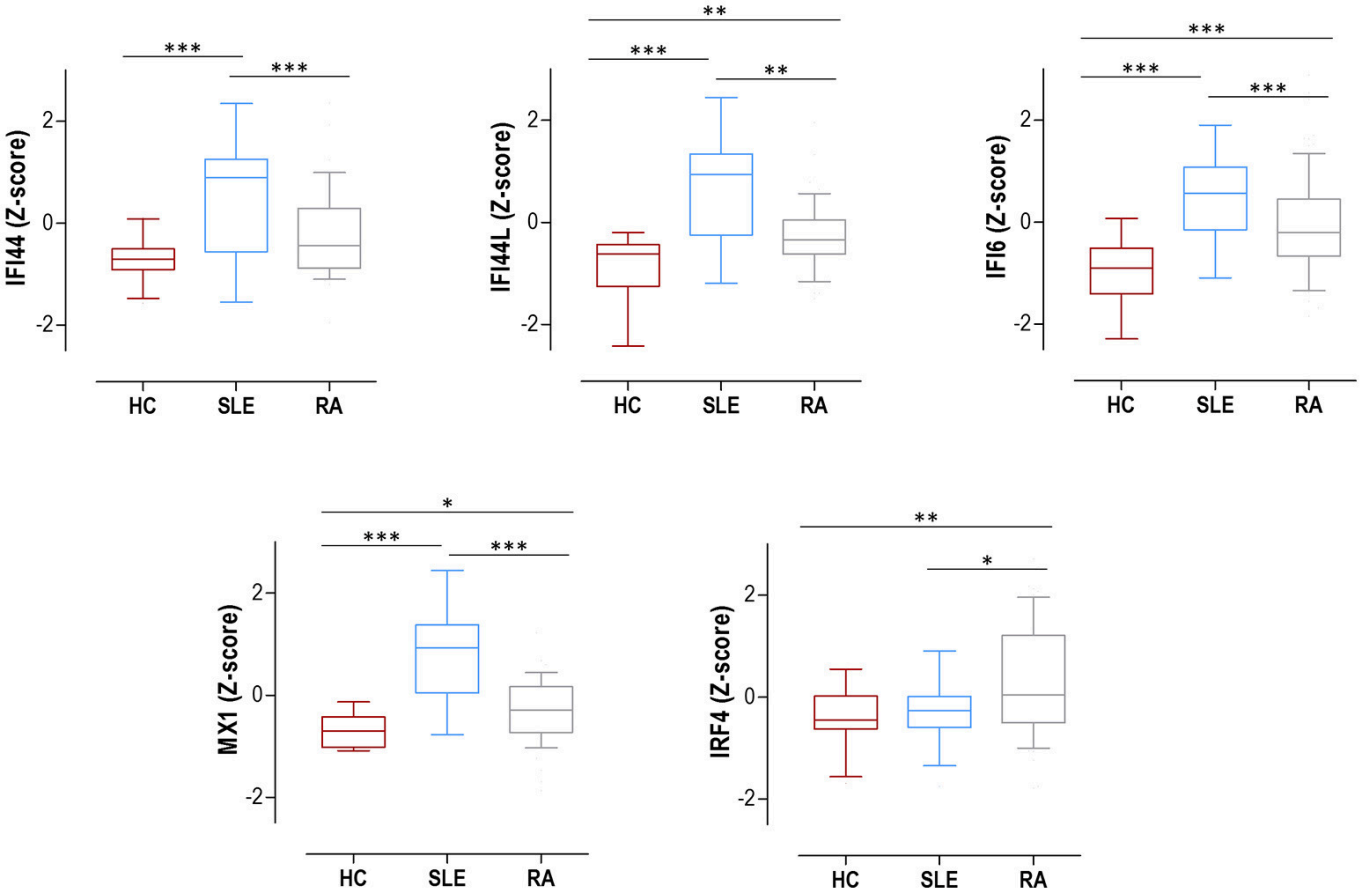
Expression of IRF4 and IRGs in SLE, RA patients and HC. IRF4 and IRGs (IFI44, IFI44L, IFI6, and MX1) gene expression in HC (dark red boxes) (*n* = 28), SLE patients (blue boxes) (*n* = 75), and RA patients (gray boxes) (*n* = 98). Results are shown as box plots, where the boxes represent the 25th and 75th percentiles, the lines within the boxes representing the median, and the lines outside the boxes represent the minimum and maximum values. Differences were assessed by Kruskal-Wallis with Dunn-Bonferroni tests for multiple comparisons. *P*-values correspond to those obtained in the multiple comparisons tests and are indicated as follows: *p < 0.050, **p < 0.010, and ***p < 0.001.

The expression of IRF4 and four IRGs (IFI44, IFI44L, IFI6, and MX1) was quantified in 75 SLE patients (Supplementary Table 1), 98 RA patients (Supplementary Table 2) and 28 HC. All IRGs were increased in autoimmune patients, to a higher degree in SLE (Figure 1). IRF4 expression was found to be increased in RA patients compared to both SLE patients and HC (Figure 1). No differences on IRF4 expression by seropositivity status were found in RA patients (Supplementary Figure 1).

To evaluate whether differences in peripheral blood cell composition may account for the differences observed in the expression of IRF4 and IRGs, multiple regression models including the frequency of monocytes, lymphocytes and neutrophils as covariables were carried out for each gene expression. None of the cell populations analyzed were related to gene expression in any condition (all *p* > 0.050, data not shown), hence ruling out the possibility that a differential cell composition underlie these findings.

All these results support a broad activation of IRGs in autoimmune patients, especially in SLE, whereas IRF4 expression was only increased in RA.

### IRF4 and IRGs Expression: Global Analysis and Cluster Approach

Based on our previous findings, we aimed to evaluate whether distinct associations between IRF4 and IRGs may underlie the differences observed among RA and SLE patients, leading to the identification of global gene expression signatures.

**Figure 2:**
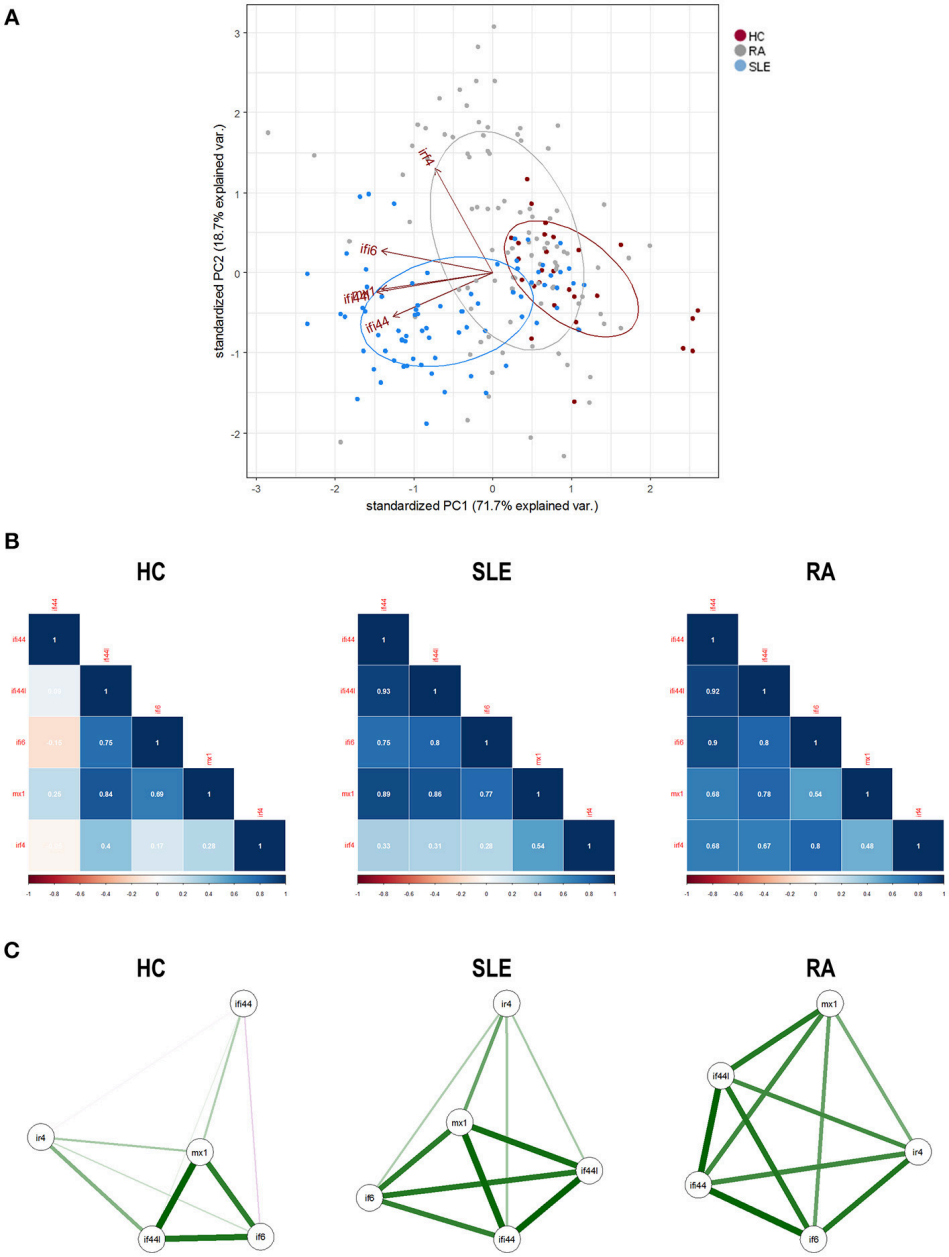
Associations among IRF4 and IRGs in autoimmune patients and HC. **(A)** Biplot originated from the PCA (correlation matrix method) conducted on the study groups recruited [HC (dark), SLE (blue), and RA (gray)]. Arrows delineate the associations among the original variables entered in the analysis (IRF4 and IRGs expression). **(B)** Analysis of the correlations among IRF4 and IRG in the different study groups. Correlation matrices were plotted in correlograms, where the color of the tiles is proportional to the strength of the correlation between each pair of genes. Correlation coefficients were depicted in white. **(C)** Network analyses depicted based on the IRF4 and IRGs expression in the different study groups. Each node corresponds to a single gene and the lines between nodes illustrate the strength (width) and type (green: positive, red: negative) of the correlations between each pair of genes. The relative position of the nodes parallels its degree of correlation that is, nodes more closely correlated locate closer to each other. The architecture defined by IRF4 and IRGs differed among conditions and it went from a weaker structure in HC toward a more concentric and uniform network in RA. The different genes analyzed followed different grouping patterns among disease status.

First, we conducted a PCA with the IRF4 and IRGs expression. PCA revealed a good adequacy of the data (KMO = 0.741, Barlett' spherificity test *p* = 8.240·10^−208^) and identified 2 components that accounted for 90.43% of the total variance. The biplot generated (Figure 2A) showed that, although certain degree of overlap existed, different global signatures could be distinguished. Whereas, patients (both SLE and RA) exhibited a different distribution than HC regarding PC1 (horizontal axis), SLE and RA patients diverged from each other in PC2 (vertical axis), hence suggesting that the associations among genes could differ among groups. Then, the associations among IRF4 and IRGs were plotted in correlation graphs (Figure 2B). This approach confirmed that gene expression patterns were not homogenous, but different pictures can be distinguished, especially regarding the role of IRF4 and the overall degree of correlation. On the one hand, both SLE and RA exhibited a higher degree of correlation among genes than that of observed in HC. Interestingly, stronger correlations were observed for IRF4 expression in RA, whereas the same was applied to MX1 in SLE. Network graphs plotted to visualize the mutual interactions among independent genes revealed different structures among groups, IRGs following different grouping patterns and IRF4 exhibiting a different relative position depending on disease status (Figure 2C). A weaker network was observed in HC, strong correlations being only found among IFI44L, IFI6, and MX1. SLE patients exhibited strong correlations among IFI44, IFI44L, IFI6, and MX1, whereas IRF4 lay apart from this cluster of genes. A different picture was observed in RA, with a concentric network hallmarked by a higher and more uniform degree of correlation among all genes analyzed. These results strengthened our previous observations.

**Figure 3:**
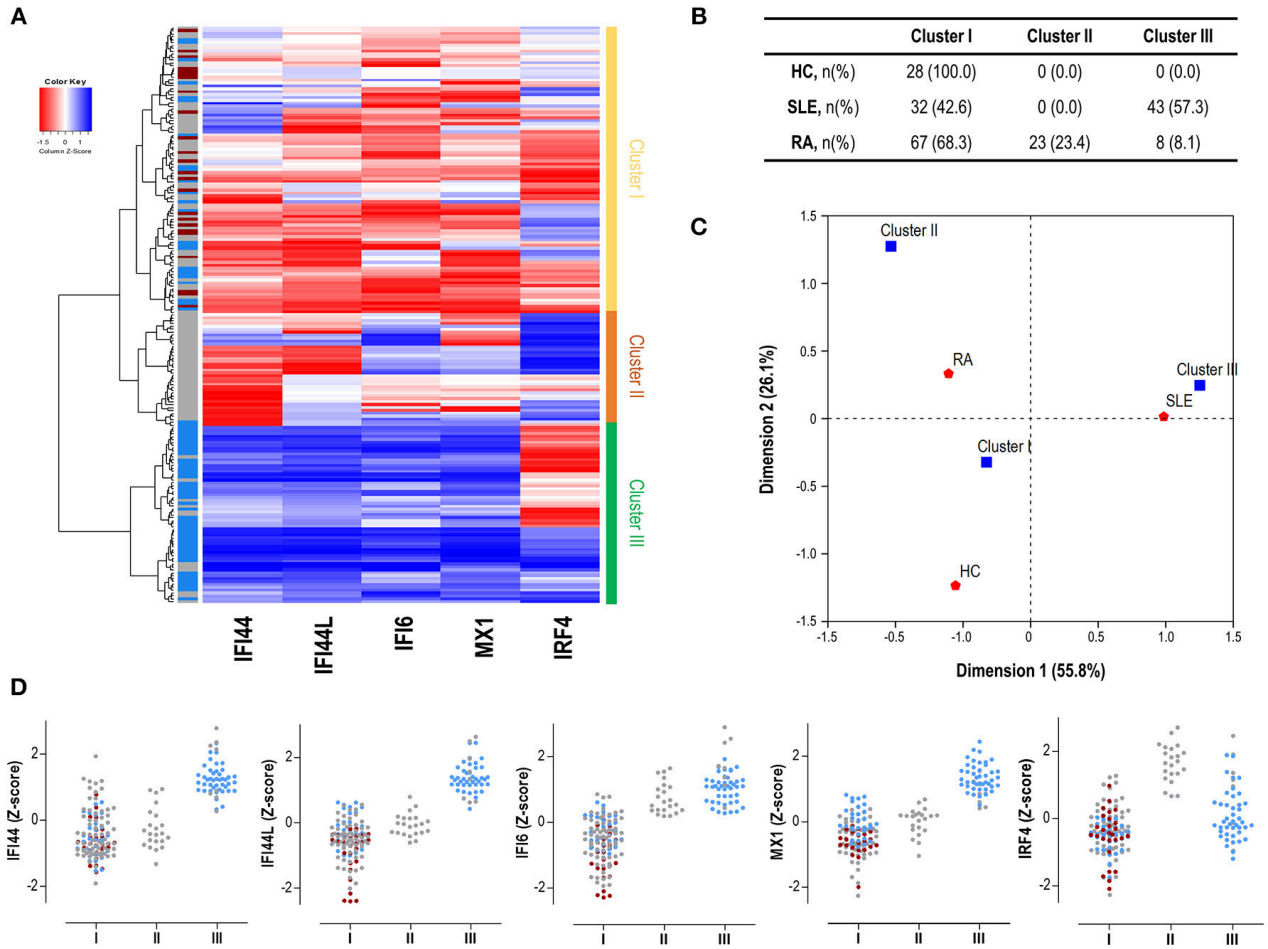
Gene signatures defined by IRF4 and IRGs. **(A)** Heatmap and cluster analysis revealing the identification of the three clusters based on the expression of IRF4 and IRGs (columns). Each row represents a study subject. Colors in the vertical left bar denoted HC (dark red), SLE patients (blue), or RA patients (gray). Vertical right bar indicates the clusters identified: cluster I (yellow), cluster II (orange), and cluster III (green). Tiles are colored based on gene expression levels, red and blue indicating low or high levels, respectively, as indicated in the legend (top left). **(B)** Table indicating the number of individuals of each study group (HC, SLE patients, and RA patients) using the different clusters identified. **(C)** Correspondence analysis (weighted chi-square distances) to study the associations between disease status (red signs) and the three clusters identified (blue signs). Axes represent the dimensions derived from the analysis. **(D)** Levels of expression for all the genes analyzed (IRF4 and IRGs) stratified by the clusters identified in the analysis. For each cluster, dots are colored according to disease status as follows: HC (dark red), SLE patients (blue), and RA patients (gray). Each dot represents one individual. These graphs were only included for visualization purposes since they were derived from previously identified expression profiles, based on individual gene expression levels. Then, no statistical analysis was performed.

Finally, we performed an unsupervised cluster analysis to assess whether these differences could delineate gene expression signatures related to disease status. Cluster analysis (Figure 3A) revealed 3 independent clusters: cluster I, characterized by a low expression of all genes analyzed; cluster II, characterized by a medium expression of IRGs and a high expression of IRF4, and cluster III, characterized by an enhanced expression of IRGs and a low expression of IRF4. Cluster I included all HC and some patients, cluster II only included RA patients and cluster III included mostly SLE and some RA patients. The frequency of each cluster differed by disease status (*p* < 0.001) (Figure 3B). Each disease exhibited a predominant cluster, as confirmed by a correspondence analysis (Figure 3C), hence demonstrating that SLE patients were closely related to cluster III, whereas RA patients did to cluster II. The individual expression of each gene stratified by clusters and according to disease diagnosis can be observed in Figure 3D.

All these results support that, apart from quantitative differences in gene expression, distinct associations among IRF4 and IRGs can be observed in autoimmune patients. These differences define specific expression signatures which are in turn differentially related to SLE and RA.

### IRF4 and IRGs Expression Signatures: Association With Clinical Features

Next, we analyzed whether the different gene signatures identified by cluster analysis were related to clinical features.

SLE patients exhibiting cluster III were younger at the time of diagnosis and were more likely to suffer from lupus nephritis and tended to exhibit elevated ESR than those in cluster I (Table 1). Additionally, patients in cluster III exhibited a higher prevalence of anti-SSA/Ro and anti-RNP antibodies than their cluster I counterparts (Table 1). As a consequence, the number of ENAs was higher in cluster III than in cluster I SLE patients (1.21 ± 0.91 vs. 0.58 ± 0.71, *p* = 0.002). Importantly, patients between both clusters did not differ in age (*p* = 0.114), gender (*p* = 0.582) or frequency of treatments (Table 1).

**Table 1:** Association between gene expression signatures and clinical features in SLE patients.

	**Cluster I (*n* = 33)**	**Cluster III (*n* = 42)**	***p*-value**
**DISEASE FEATURES**			
Disease duration, years; median (range)	12.12 (0.33–39.00)	14.75 (0.17–32.00)	0.222
Age at diagnosis, years; median (range)	38.10 (18 - 68)	28.50 (19 - 65)	0.023
ESR, mm/h	10.50 (10.25)	14.50 (12.75)	0.071
Disease activity (SLEDAI)	3.50 (5.00)	2.00 (3.58)	0.769
**CLINICAL MANIFESTATIONS**, ***N*****(%)**			
Malar rash	16 (48.4)	24 (57.1)	0.456
Discoid lesions	10 (31.2)	7 (16.6)	0.161
Photosensitivity	17 (51.1)	24 (57.1)	0.627
Oral ulcers	16 (48.4)	24 (57.1)	0.456
Arthritis	22 (66.6)	29 (69.0)	0.826
Serositis	8 (24.2)	9 (21.4)	0.773
Cytopenia	24 (72.7)	27 (64.2)	0.437
Lupus nephritis	5 (15.1)	17 (40.4)	0.021
Neurological disorder	3 (9.0)	5 (11.9)	0.695
**AUTOANTIBODIES**, ***N*****(%)**			
ANA	33 (100)	42 (100)	–
Anti-dsDNA	28 (84.4)	32 (76.1)	0.352
Anti-SSA/Ro	12 (39.3)	28 (66.6)	0.009
Anti-SSB/La	4 (12.1)	8 (19.0)	0.417
Anti-Sm	1 (3.0)	5 (11.9)	0.160
Anti-RNP	1 (3.0)	10 (23.8)	0.012
Anti-RibP	3 (9.0)	6 (14.2)	0.522
RF	5 (15.1)	8 (19.0)	0.319
**TREATMENTS**, ***N*****(%)**			
None	1 (3.0)	2 (4.7)	–
Glucocorticoids	14 (42.4)	15 (35.7)	0.328
Antimalarials	27 (81.1)	39 (92.8)	0.720
Mycophenolate mophetil	0 (0)	1 (2.3)	-

*Variables were expressed as median (interquartile range) or n(%), unless otherwise stated. Differences were assessed by Mann-Withney or chi-square tests (or Fisher exact test, when appropriate), according to the distribution of the variables*.

On the other hand, RA patients were distributed among the 3 clusters identified. A differential distribution of the patients according to disease stages was noted, since the frequency of the VERA group (recruited at onset, untreated) was enriched in cluster I (Table 2). Interestingly, lower disease activity score, joint involvement and ESR was observed in cluster II (Table 2). No differences in gender (*p* = 0.393), age (*p* = 0.721) and treatment usage (Table 2) were found. These results were maintained after excluding VERA patients from the analysis (Supplementary Table 3), thus suggesting a milder course of RA patients using cluster II, since cluster I patients were hallmarked by a more active disease despite being more intensively treated than their cluster II-counterparts. Although higher ESR and frequency of autoantibodies was found in cluster III RA patients, the low sample size observed was insufficient to drawn firm conclusions.

**Table 2:** Association among gene expression signatures and clinical features in RA patients.

	**Cluster I (*n* = 67)**	**Cluster II (*n* = 23)**	**Cluster III (*n* = 8)**	***p*-value**
**DISEASE FEATURES**				
Disease duration, years; median (range)	3.80 (0–30.00)	4.91 (0.17–20.00)	5.37 (1.75–16.25)	0.360
Age at diagnosis, years; median (range)	46.29 (23 - 62)	49.16 (19 - 61)	50.33 (18 - 65)	0.968
ESR, mm/h	21.50 (29.50)	10.50 (19.00)	37.50 (36.25)	0.025^a^
Disease activity (DAS28)	4.40 (2.08)	3.10 (1.94)	3.76 (3.02)	<0.001^b^
Tender Joint Count	3.00 (5.00)	1.00 (2.00)	0.00 (5.00)	0.019^c^
Swollen Joint Count	4.00 (9.00)	1.00 (3.50)	2.50 (5.25)	0.004^d^
Patient global assessment (0–100)	50.00 (34.00)	25.00 (40.00)	50.00 (41.25)	0.028^e^
Pain assessment (0–10)	5.00 (3.65)	2.00 (5.00)	4.50 (4.75)	0.020^f^
HAQ (0–3)	1.12 (0.92)	0.50 (1.25)	0.50 (1.41)	0.020^g^
**AUTOANTIBODIES**, ***N*****(%)**				
RF	41 (61.2)	12 (52.1)	5 (62.5)	0.645
ACPA	40 (59.7)	14 (60.8)	6 (75.0)	0.411
RF or ACPA	44 (65.5)	15 (65.0)	7 (87.5)	0.641
RF and ACPA	31 (46.2)	10 (43.4)	5 (62.5)	0.229
**TREATMENTS**, ***N*****(%)**				
None (VERA)	16 (23.8)	1 (4.3)	0 (0.0)	0.047
Glucocorticoids	41 (61.2)	9 (39.1)	5 (62.5)	0.286
Methotrexate	41 (61.2)	17 (73.9)	7 (87.5)	0.130
TNFα blockers	24 (35.8)	8 (34.7)	4 (50.0)	0.773

*Variables were expressed as median (interquartile range) or n(%), unless otherwise stated. Differences were assessed by Kruskal-Wallis or chi-square tests (or Fisher exact test, when appropriate), according to the distribution of the variables. The p-values in the table correspond to the Kruskal-Wallis or chi-square tests. Multiple comparisons tests (Dunn-Bonferroni correction) were performed when the Kruskal-Wallis test revealed significant differences among groups and p-values were summarized in superscripts*.

a *I vs. II: p = 0.080, II vs. III: p = 0.032, I vs. III: p = 0.409*.

b *I vs. II: p < 0.001, II vs. III: p = 0.070, I vs. III: p = 0.497*.

c *I vs. II: p = 0.003, II vs. III: p = 0.433, I vs. III: p = 0.574*.

d *I vs. II: p = 0.043, II vs. III: p = 0.841, I vs. III: p = 0.233*.

e *I vs. II: p = 0.020, II vs. III: p = 0.518, I vs. III: p = 0.910*.

f *I vs. II: p = 0.009, II vs. III: p = 0.438, I vs. III: p = 0.774*.

g *I vs. II: p = 0.028, II vs. III: p = 0.790, I vs. III: p = 0.443*.

In sum, gene expression profiles defined by IRF4 and IRGs expression are associated with clinical features of severity in SLE patients as well as with disease activity and clinical stage in RA. Additional and larger studies are warranted to shed some light into their potential clinical implications as a biomarker.

### IRF4 and IRGs Expression Upon TNFα-Blockade

In order to get insight into the IRF4 expression and its association with that of IRGs upon TNFα-blockade, as well as its potential relevance as a biomarker of therapy outcome, the IRF4 and IRGs expression was prospectively analyzed in a subgroup of 13 biological-naïve RA patients at baseline (BL) and after three months upon TNFα-blockade (post-treatment, PT).

**Figure 4:**
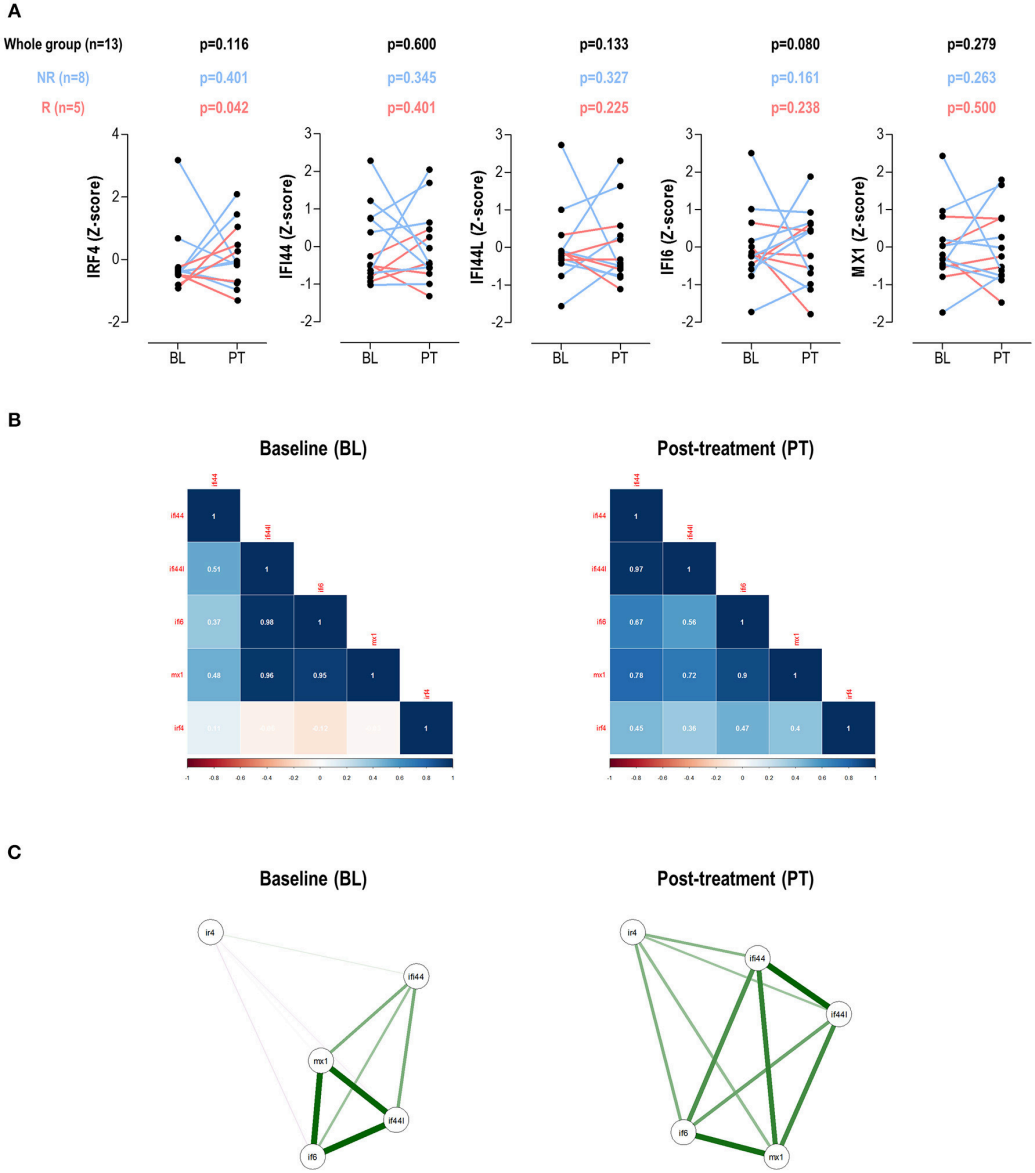
Changes in IRF4 and IRGs expression upon TNFa-blockade. **(A)** Paired analyses (Wilcoxon tests) of the IRF4 and IRGs expression at baseline (BL) and post-treatment (PT) upon TNFα-blockade in a group of 13 biological-naïve RA patients prospectively followed up. Patients were denoted in red (responders) and blue (moderate/non-responders). The *p*-values show on top of the graphs were derived from the analysis of the whole patient population (*n* = 13, black numbers), responders (*n* = 5, red numbers), or non-responders (blue, *n* = 8). **(B)** Correlation plots and network analyses **(C)** of the IRF4 and IRGs expression in BL and PT samples.

No changes in IRF4 expression, neither in IRGs, were detected in the whole group upon TNFα-blockade (Figure 4A). IRF4 expression did not correlate DAS28 at BL (*r* = −0.088, *p* = 0.775) nor PT (*r* = 0.306, *p* = 0.310). No changes in leukocytes, neutrophils, lymphocytes or monocytes counts were observed upon treatment (all *p* > 0.050, data not shown). When patients were stratified by treatment response, increasing IRF4 expression upon treatment was observed in responders compared to their non-responder counterparts (Figure 4A). No difference in IRF4 at baseline was observed between groups (*p* = 0.464).

Next, the associations among IRF4 and IRGs upon TNFα-blockade were studied. Correlation plots revealed clear differences among these genes between baseline and post-treatment samples (Figure 4B). Additionally, the network analyses (Figure 4C) confirmed changes in the correlation profiles among IRF4 and the IRGs. Stronger associations among IFI44L, IFI6, and MX1 were found in the BL samples, with weak or no associations with IRF4. This picture partially mirrored that of observed in SLE patients in the cross-sectional analysis. Interestingly, a more uniform pattern among all genes was observed after treatment, similar to that of the RA patients in the cross-sectional study, hence suggesting distinct gene expression programs before and after TNFα-blockade.

Taken together, these results confirm that changes in IRF4 expression are associated with therapy outcomes upon TNFα-blockade in the short-term (3 months). Moreover, the associations among genes largely differed before and after treatment, hence confirming qualitative changes in the gene expression program in this scenario. These findings warrant further studies to elucidate the relevance of these changes in the long-term.

### Validation in Public Microarrays Datasets

Finally, data of IRF4 expression in peripheral blood in autoimmune patients was extracted from publicly available microarray datasets downloaded from the GEO database in order to validate our results. Five datasets containing relevant samples were retrieved: 4 datasets analyzing RA patients (1 in peripheral blood and 3 in synovial tissue) and 1 dataset including multiple sclerosis (MS) patients (peripheral blood).

**Figure 5:**
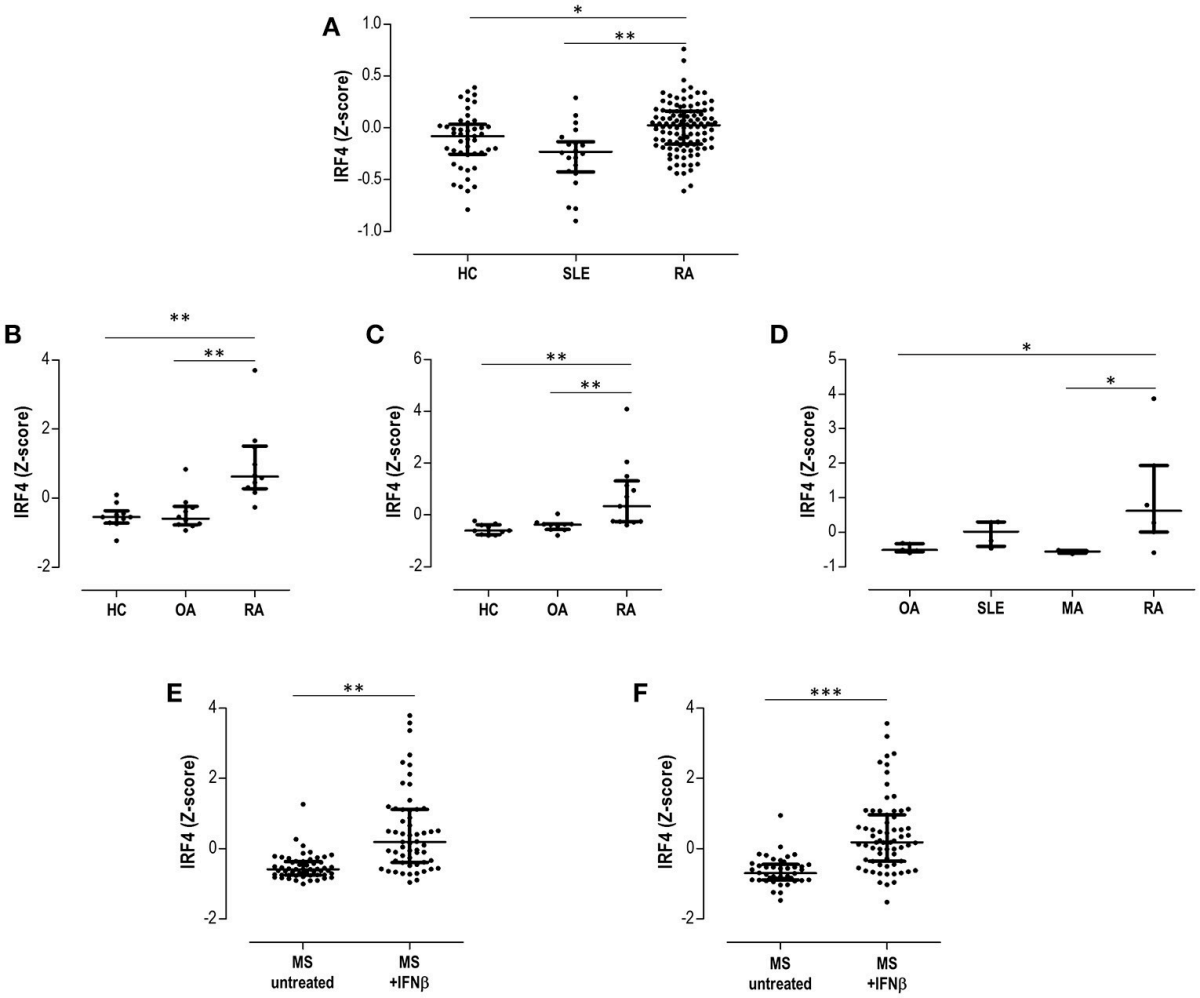
Validation in publicly available GEO datasets. **(A)** Expression of IRF4 in peripheral blood in HC, SLE patients and RA patients extracted from the dataset GSE17755. The differential expression of IRF4 in synovial membranes from RA patients was confirmed in datasets GSE55457 **(B)**, GSE55235 **(C)**, and GSE36700 **(D)**. The expression of IRF4 in MS patients under IFNb treatment was evaluated in the dataset GSE41846 in cross-sectional **(E)** and prospective **(F)** samples. Expression data from each dataset were extracted and Z-scores were calculated and plotted in scatter dot plots. Each dot represents one individual and bars represent median values. Upper and lower whiskers represent the 75th and 25th values, respectively. IRF4 was confirmed to be differentially expressed in each dataset with the GEO2R tool, as indicated in the Results section. Statistical analysis on graphs was performed by conventional tests (Kruskal-Wallis or Mann-Withney *U*-tests, as appropriate). The *p*-values are indicated as follows: *p < 0.050, **p < 0.010, and ***p < 0.001.

First, GSE17755 included gene expression data from 45 HC, 22 SLE, and 112 RA patients. IRF4 was found to be differentially expressed by GEO2R (adjusted *p* = 3.13·10^−4^) among groups, increased expression being confirmed in RA (Figure 5A). Next, in order to gain additional insight on the IRF4 expression in RA, datasets containing gene expression data from target tissues (synovial membrane) were analyzed. GSE55457 included gene expression data from 10 HC, 10 osteoarthritis (OA) patients and 13 RA patients. IRF4 was observed to be differentially expressed among patients (adjusted *p* = 6.40·10^−4^), being upregulated in RA (Figure 5B). An equivalent result was obtained from GSE55235 (10 HC, 10 OA, and 10 RA) (adjusted *p* = 1.04·10^−4^) (Figure 5C). Results from GSE36700 containing synovial tissue samples from 5 OA, 4 SLE, 5 microcrystalline arthritis (MA) and 7 RA patients confirmed the differential expression of IRF4 (adjusted *p* = 8.4·10^−4^), again being upregulated in RA (Figure 5D). Finally, data on IRF4 expression was analyzed in other autoimmune diseases. GSE41846 contained gene expression data from a cross-sectional study on 54 untreated and 57 IFNβ-treated multiple sclerosis (MS) patients. IRF4 was found to be differentially expressed (adjusted *p* = 3.63·10^−8^), being upregulated in IFNβ-treated patients (Figure 5E). The same dataset contained follow up data (longitudinally collected at 1 year visit) from 42 untreated MS patients and 67 IFNβ-treated MS patients supporting the increased IRF4 expression (adjusted *p* = 1.59·10^−7^) (Figure 5F).

Taken together, these analyses confirmed the differential expression of IRF4 in autoimmune patients, being increased in RA patients both in peripheral blood and in peripheral tissues, as well as in MS after IFNβ treatment.

## Discussion

Despite the type I IFN signature being widely recognized as a common mediator in several systemic autoimmune diseases, a precise definition of its components and regulatory mechanisms is still lacking. Indeed, some authors have highlighted that distinct mediators may be differentially associated with the type I IFN signature(s) in different diseases. The findings herein presented shed new light on the role of a new factor, the IRF4, as a new player in this scenario. A differential activation of the IRF4 expression together with distinct associations with IRGs define global expression signatures in SLE and RA patients with clinical relevance. Taken together, our results expand the notion of the IFN signature shaping by IRFs in autoimmune diseases.

A major breakthrough of our study was the characterization of different gene expression signatures driven by IRF4 and IRGs. The differential associations among these genes delineate distinct gene interactions that can provide additional information to better understand the structure of the IFN signature in different diseases. Although early studies only considered the type I IFN signature as a sole measure of IRGs activation, recent studies have stressed the need of more complex approaches. In this sense, Reynier and colleagues (46) demonstrated that the overall state of correlation must be also considered when analyzing genes with similar expression patterns, such as the type I IFN signature in order to account for co-regulation and co-expression phenomena. Using an equivalent approach, we have found that beyond their differential expression, the figures of IRF4 and IRGs differed in terms of their mutual associations among conditions, hence adding another layer of complexity to the analysis of the type I IFN signature. In fact, SLE and RA patients clearly differed in the associations observed between IRF4 and the IRGs, thus pointing to distinct molecular programs hallmarking these two conditions.

The heterogeneity of the type I IFN signature has been largely debated during last years. Data suggest that heterogeneity in the type I IFN signature activation and genetic make-up contribute to the clinical heterogeneity observed in rheumatic conditions (7), thus underlining the need of a better understanding of the IFN signature architecture. Recently, de Jong and colleagues have found that certain diversification of the type I IFN signature can be recognized among different diseases (39). More importantly, this diversification has been suggested to parallel the clinical course of some patients (37, 39, 41). In a similar way, we have reported that different profiles can be distinguished in the type I IFN signature of RA patients according to their clinical stage (36). As a consequence, this body of evidence strongly supports that the type I IFN signature may be more complex than initially thought and its fine structure emerges as a relevant topic. In the present study, we went further by defining gene signatures related to the type I IFN signature and IRF4 expression, which were differentially used by different autoimmune diseases. These gene expression programs identified subsets of patients with distinct clinical features, hence strengthening their clinical relevance. The fact that different gene signatures are linked to specific clinical features within a single disease may account for the controversy observed at the disease level among studies and may help to dissect the molecular complexity of these conditions. Furthermore, these differences were observed to correlate with clinical phenotypes. As a consequence, our findings provide a rationale to include the IRF4 in future studies assessing the type I IFN signature in autoimmune patients to resolve the heterogeneity of the IFN signature as well as to gain additional insight into the differential architecture of the IFN signature in these complex diseases.

The distinct, mutual expression patterns related to IRF4 and IRGs expression in SLE and RA patients support the concept that IRFs could play a role in the regulation and editing of the type I IFN signature in autoimmunity. Although previously restricted to IRF3 and IRF7, our findings expand the notion that IRFs may be associated with the modulation of IRGs toward new family members, the IRF4. IRF4 has been reported to be a negative regulator of the TLR pathway (32, 47), hence leading to a decreased expression of a number of IRGs *in vitro*. Interestingly, IRF4 has been demonstrated to interact with MyD88, a molecular hub for the control of IRGs expression (32). More importantly, IRF4 competes with IRF5 for MyD88 interaction, and IRF4 expression leads to the inhibition of IRF5-dependent genes. Accordingly, gene expression profiles from Irf4^−/−^ macrophages mirrored that of their Irf5^−/−^ counterparts (32). Furthermore, a recent paper by Forero and coworkers has added new clues to the relationship of IRF4 and IRGs (31). Using an inducible expression system, it has been demonstrated that IRF4 acts as a regulator of IRGs induction, different subsets of IRGs being identified: from positively (such as ISG60 or OAS) to negatively regulated IRGs (MX1). Additionally, the IRGs differ in terms of their sensitivity to the IRF4-mediated modulation, which may be caused by a different affinity of IRF4 for Interferon-sensitive response element (ISRE) sites in such IRGs (47). Taken together, these lines of evidence underline the role of IRF4 in shaping the type I IFN signature. However, the clinical relevance of these findings remained unknown. The findings herein presented shed new light into the clinical value of this complex regulation between IRF4 and IRGs in autoimmune patients and prove this field worthy of further research in order to better delineate the effect of IRFs on the IFN signature(s) in autoimmunity.

In addition to deciphering new interactions within the IFN signature in systemic autoimmune conditions, our results are relevant from a translational point of view. First, the global analysis of IRF4 and IRGs allow us to identify a subset of SLE patients with clinical features of disease severity and enhanced autoantibody production (cluster III). Interestingly, RA patients exhibiting the same gene expression signature mirrored such clinical characteristics. Moreover, RA patients showing the gene signature characterized by a high IRF4 expression exhibited a low-grade clinical phenotype, hence pointing to a connection between IRF4 expression and mild disease course and/or a better response to therapies. This notion was supported by our results from the prospective analysis in patients undergoing TNFα-blockade, increasing IRF4 expression being related to a good clinical outcome. Further follow up studies to elucidate the long-term clinical relevance of these findings are warranted.

Despite the fact that clusters defined by IRF4 and IRGs were related to disease activity/severity, the patient populations were overall related to a mild disease activity. This was especially clear in SLE patients, since patients exhibited a good control of the disease despite the scarce use of strong immunosuppressants or biological drugs. However, our experimental approach was able to identify a disease subset with markers of severity and poor prognosis (increased nephritis and autoantibodies), regardless of disease activity. Whether the lack of differences in the SLEDAI score may be attributed to the overall low disease activity of the SLE patients cannot be totally excluded. However, since disease activity fluctuates, the IRF4/IRGs system may be a more reliable marker of severity in these patients. In the case of RA patients, both IRF4 expression level (elevated in cluster II patients and increasing levels in TNFα-blockade responders) and its associations with IRGs was associated with disease activity, hence adding another layer of complexity to the clinical relevance of the IFN signature structure in this condition.

The different pictures observed for the type I IFN signature in SLE and RA, especially regarding IRF4 activation, are relevant to understand unresolved questions from previous studies. On the one hand, Smiljanovic and colleagues demonstrated that the IFN signature observed in RA patients qualitatively differs from that of their SLE counterparts in terms of target genes and transcription factors binding sites, and, remarkably, genomic imprints found in RA patients were more heterogeneous (48). The differential upregulation of IRF4 between these two conditions together with the usage of the 3 gene clusters in RA compared to only 2 being observed in SLE is line with these findings, hence reinforcing the role of IRF4 in this setting. Additionally, IFNβ has been reported to contribute to the global type I IFN signature in RA (49), whereas other systemic conditions, such as SLE, are thought to be mostly IFNα-driven (39). Interestingly, our results support a link between IFNβ and IRF4 upregulation. Hence, IRF4 emerges as a pivotal player to understand the divergences in the IFN signature among conditions from a mechanistic perspective. Additionally, the protective effects of IFNβ in RA (50, 51) align with the mild clinical course of RA patients with elevated IRF4 expression. Consequently, it may be conceivable that IRF4 could be regarded as a pharmacodynamic clinical biomarker for IFNβ treatment in RA patients, a major unmet need that limits the application of this therapy. Recently, increased serum IFN-β/α ratio activity in RA patients has been demonstrated to predict poor clinical outcome upon TNF inhibition (52). Unfortunately, important methodological differences limit the interpretation of our findings in the light of the results reported by Wampler and coworkers. Finally, Gordon and colleagues found that the IFN signature in RA can be also influenced by TNF (53), thus supporting that mediators other than type I IFNs contribute to this expression program in RA patients. Interestingly, IRF4 has been linked to the NFkB pathway (31, 54, 54), which is central for RA pathogenesis. Since the NFkB pathway is activated by TNF signaling, IRF4 could be an important mediator to understand the TNF-related type I IFN signature upregulation in RA. Being associated with a milder course and clinical response to TNFa-blockade, it is tempting to speculate that IRF4 upregulation may be considered as a therapeutic opportunity in RA. However, its functional association with NFkB pathway needs to carefully considered in this setting. As a consequence, the potential conception of IRF4 as a therapeutic target warrants further investigation.

In conclusion, our results revealed that distinct levels of expression and differential associations of IRF4 with IRGs may identify gene expression signatures with clinical relevance in SLE and RA patients. To the best of our knowledge, this is the first study analyzing the IRF4 expression in peripheral blood in autoimmune patients and its association with IRGs expression as well as with clinical features and treatment outcomes. Therefore, this proof-of-concept study sheds new light on the structure of type I IFN signature and support a role for IRF4 as potential modulator with clinical added value. A number of potential limitations of the present study must be acknowledge. First, patient populations were not fully comparable in terms of disease duration, as expected from distinct clinical entities with different age at onset. Moreover, a mild clinical course was observed in the SLE population, whereas an overall low degree of activity was found in RA patients. Although clear associations between IRF4 and activity/severity were retrieved, future studies including patients with very high disease activity would be advisable to further confirm our results. Finally, a partial number of IRGs and only IRF4 (among all IRFs family members) were included in this study. Whether the IFN signature shaping can be extended to other IRFs remains unknown and warrants further studies. Therefore, the findings herein presented pave the ground for future, larger studies involving a higher number of IRFs and IRGs in different autoimmune conditions.

## Author Contributions

JR-C carried out most of the experimental procedures, performed the statistical analyses and drafted and edited the manuscript. PL performed some experimental procedures. MA-L, LC-M, and FB-G were in charge of patients' recruitment and clinical data management. AS conceived the study, designed the protocols and drafted and edited the manuscript. All authors read and approved the final version of the manuscript.

### Conflict of Interest Statement

The authors declare that the research was conducted in the absence of any commercial or financial relationships that could be construed as a potential conflict of interest.

## Abbreviations

ACR: American College of Rheumatology
ACPA: anti-citrullinated peptide antibodies
ANA: antinuclear antibodies
csDMARDs: conventional synthetic DMARDs
DAS28: disease activity score 28-joints
DMARDs: disease-modifying antirheumatic drugs
ENA: extractable nuclear antigens
EULAR: European league against rheumatism
ESR: erythrocyte sedimentation rate
HAQ: health assessment questionnaire
HC: healthy controls
IFI6: interferon alpha inducible protein 6
IFI44: interferon induced protein 44
IFI44L: interferon induced protein 44 like
IFN: interferon
IRF: interferon regulatory factor
IRG: interferon-responding gene
MX1: MX dynamin like GTPase 1
NSAIDs: non-steroidal anti-inflammatory drugs
RA: rheumatoid arthritis
RF: rheumatoid factor
SLE: systemic lupus erythematosus
RNP: ribonucleoproteins
RibP: ribosomal P protein
SLEDAI: systemic lupus erythematosus disease activity index
TNFα: tumor necrosis factor alpha.
