# Supplementary material for: IRF4 and IRGs Delineate Clinically Relevant Gene Expression Signatures in Systemic Lupus Erythematosus and Rheumatoid Arthritis
Source: Front Immunol. 2019 Jan 7;9:3085. doi: 10.3389/fimmu.2018.03085 (PMC6330328; doi:10.3389/fimmu.2018.03085)
Supplement: Supplementary file 1 [file Table_2.DOCX]

**ONLINE SUPPLEMENTARY MATERIAL**

**Supplementary Table 1: Detailed clinical description of SLE patients recruited for this study.** Variables were expressed as median (interquartile range) or n(%), unless otherwise stated. Differences were assessed by Kruskal-Wallis or chi-square tests, according to the distribution of the variables.

|  | **SLE**  **(n=75)** |
| --- | --- |
| ***Disease features*** |  |
| Disease duration, years; median (range) | 13.75 (0.17 – 39.00) |
| Age at diagnosis, years; median (range) | 32.30 (18 – 68) |
| ESR, mm/h | 18.00 (29.00) |
| Disease activity (SLEDAI) | 2.00 (2.00) |
| Clinical manifestations, n(%) |  |
| Malar rash | 40 (53.3) |
| Discoid lesions | 17 (22.6) |
| Photosensitivity | 41 (54.6) |
| Oral ulcers | 40 (53.3) |
| Arthritis | 51 (68.0) |
| Serositis | 17 (22.6) |
| Cytopenia | 51 (68.0) |
| Lupus nephritis | 22 (29.3) |
| Neurological disorder | 8 (10.6) |
| ***Autoantibodies, n(%)*** |  |
| ANA | 75 (100) |
| Anti-dsDNA | 60 (80.0) |
| Anti-SSA | 40 (53.3) |
| Anti-SSB | 12 (16.0) |
| Anti-Sm | 6 (8.0) |
| Anti-RNP | 11 (14.6) |
| Anti-RibP | 9 (12.0) |
| RF | 13 (17.3) |
| ***Treatments, n(%)*** |  |
| None | 3 (4.0) |
| Glucocorticoids | 29 (38.6) |
| Antimalarials | 66 (88.0) |
| Mycophenolate mophetil | 1 (1.3) |

**Supplementary Table 2: Detailed clinical description of RA patients recruited for this study.** Variables were expressed as median (interquartile range) or n(%), unless otherwise stated. Differences were assessed by Kruskal-Wallis or chi-square tests, according to the distribution of the variables.

|  | **RA**  **(n=98)** |
| --- | --- |
| ***Disease features*** |  |
| Disease duration, years; median (range) | 4.00 (0.00 – 30.00) |
| Age at diagnosis, years; median (range) | 47.37 (19 – 65) |
| ESR, mm/h | 18.00 (29.00) |
| Disease activity (DAS28) | 2.00 (2.00) |
| Tender Joint Count | 3.00 (7.00) |
| Swollen Joint Count | 2.00 (5.00) |
| Patient Global Assessment (0-100) | 50.00 (35.00) |
| Pain Assessment (0-10) | 4.85 (3.40) |
| HAQ (0-3) | 1.00 (1.16) |
| ***Autoantibodies, n(%)*** |  |
| RF | 58 (59.1) |
| ACPA | 61 (62.2) |
| ***Treatments, n(%)*** |  |
| None | 18 (18.3) |
| Glucocorticoids | 55 (56.1) |
| Methotrexate | 65 (66.3) |
| TNFα blockers | 36 (36.7) |

**Supplementary Table 3: Association among gene expression signatures and clinical features in established RA patients.** Variables were expressed as median (interquartile range) or n(%), unless otherwise stated. Differences were assessed by Kruskal-Wallis or chi-square tests (or Fisher exact test, when appropriate), according to the distribution of the variables. The p-values in the table correspond to the Kruskal-Wallis or chi-square tests. Multiple comparisons tests (Dunn-Bonferroni correction) were performed when the Kruskal-Wallis test revealed significant differences among groups and p-values were summarized in superscripts. ^a^ I vs II: p=0.050, II vs III: p=0.132, I vs III: p=0.409; ^b^ I vs II: p=0.008, II vs III: p=0.080, I vs III: p=0.850; ^c^ I vs II: p=0.011, II vs III: p=0.276, I vs III: p=0.409; ^d^ I vs II: p=0.041, II vs III: p=0.830, I vs III: p=0.452; ^e^ I vs II: p=0.024, II vs III: p=0.324, I vs III: p=0.444; ^f^ I vs II: p=0.032, II vs III: p=0.687, I vs III: p=0.171.

|  | **Cluster I (n=51)** | **Cluster II (n=22)** | **Cluster III (n=8)** | **p-value** |
| --- | --- | --- | --- | --- |
| ***Disease features*** |  |  |  |  |
| Disease duration, years; median (range) | 6.37 (0.50 – 30.00) | 5.45 (0.92 – 20.00) | 5.37 (1.75 – 16.25) | 0.929 |
| Age at diagnosis, years; median (range) | 45.33 (23 – 62) | 49.91 (21 – 65) | 50.33 (18 – 65) | 0.616 |
| ESR, mm/h | 17.00 (27.25) | 13.00 (19.00) | 37.50 (36.25) | 0.047^a^ |
| Disease activity (DAS28) | 4.20 (1.98) | 3.18 (2.08) | 3.76 (2.02) | 0.010^b^ |
| Tender Joint Count | 3.00 (8.50) | 1.00 (1.75) | 0.00 (5.00) | 0.014^c^ |
| Swollen Joint Count | 2.00 (5.00) | 0.50 (1.75) | 2.50 (5.25) | 0.082 |
| Patient Global Assessment (0-100) | 50.00 (35.00) | 22.50 (40.00) | 50.00 (41.25) | 0.040^d^ |
| Pain Assessment (0-10) | 5.00 (4.00) | 2.00 (4.75) | 4.50 (4.75) | 0.029^e^ |
| HAQ (0-3) | 1.12 (0.82) | 0.37 (1.15) | 0.50 (1.41) | 0.021^f^ |
| ***Autoantibodies, n(%)*** |  |  |  |  |
| RF | 38 (74.5) | 13 (59.0) | 5 (62.5) | 0.6531 |
| ACPA | 31 (60.7) | 12 (54.5) | 6 (75.0) | 0.503 |
| RF or ACPA | 34 (66.6) | 14 (63.6) | 7 (87.5) | 0.753 |
| RF and ACPA | 27 (52.9) | 8 (36.3) | 5 (62.5) | 0.341 |
| ***Treatments, n(%)*** |  |  |  |  |
| Glucocorticoids | 41 (80.3) | 9 (40.9) | 5 (62.5) | 0.003 |
| Methotrexate | 41 (80.3) | 17 (77.2) | 7 (87.5) | 0.903 |
| TNFα blockers | 24 (47.0) | 8 (36.3) | 4 (50.0) | 0.810 |

**Supplementary figure legends**

**Supplementary Figure 1: IRF4 expression according to seropositivity status in RA patients. The** IRF4 gene expression was evaluated in RA patients according to seropositivity status: (A) RF/ACPA double positive patients (patients exhibiting both autoantibodies) compared to the rest (either seronegative patients or single ACPA or RF-positive patients), (B) RF or ACPA+ patients (either positive for RF, ACPA or both) compared to their seronegative-counterparts (negative for both autoantibodies) and (C) RF/ACPA-double negative, RF+ ACPA negative patients and RF+/ACPA+ patients. Gene expression is depicted in box plots, where the boxes represent the 25th and 75th percentiles, the lines within the boxes representing the median, and the lines outside the boxes represent the minimum and maximum values. Differences were assessed by Mann-Withney or Kruskal-Wallis tests.
