# Supplementary figures and images for: IRF4 and IRGs Delineate Clinically Relevant Gene Expression Signatures in Systemic Lupus Erythematosus and Rheumatoid Arthritis
Source: Front Immunol. 2019 Jan 7;9:3085. doi: 10.3389/fimmu.2018.03085 (PMC6330328; doi:10.3389/fimmu.2018.03085)

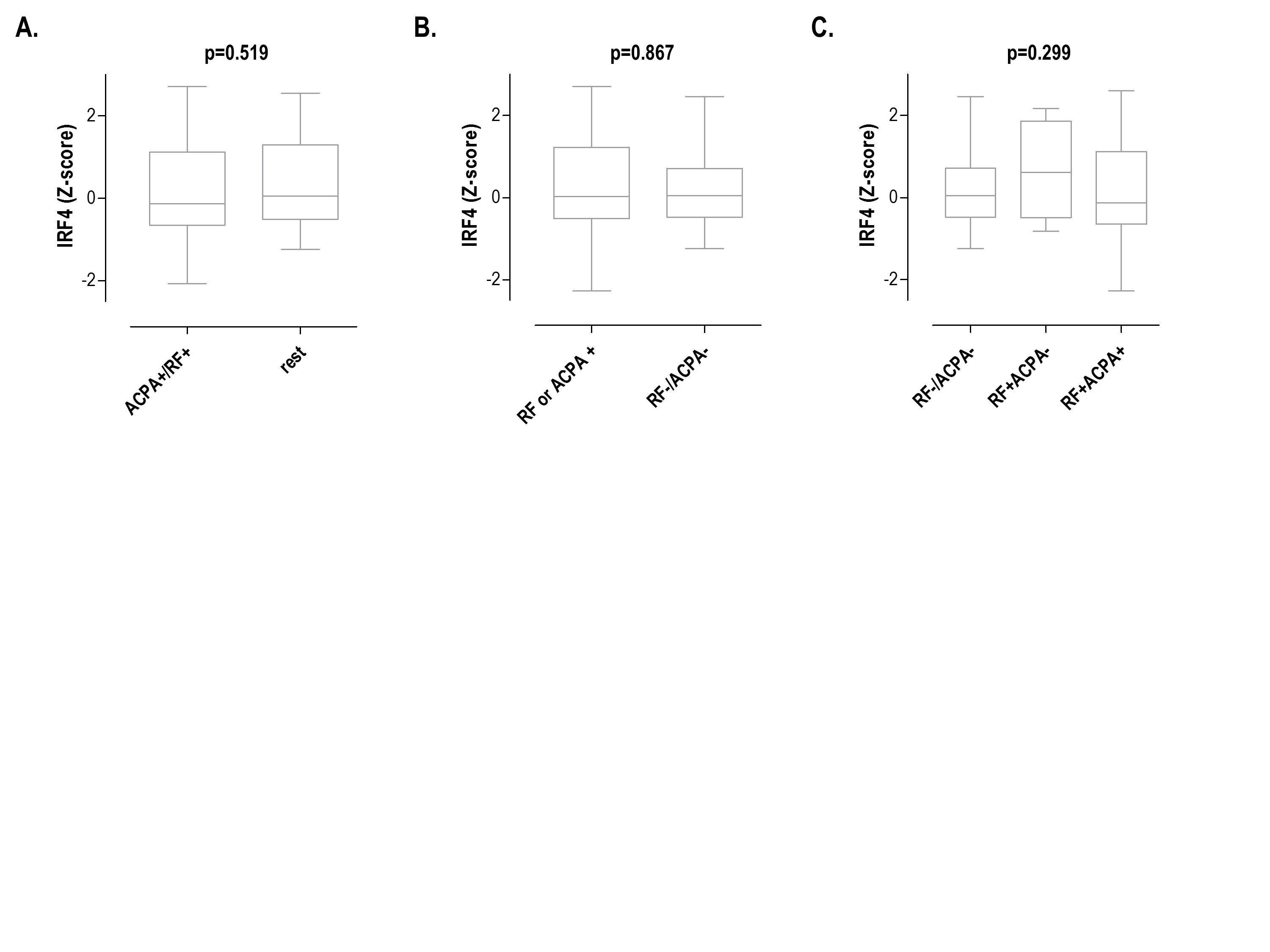

Supplement: Supplementary file 2 [file Image_1.TIF]
